# Prognostic impact of para-aortic lymph node metastases in non-pancreatic periampullary cancer

**DOI:** 10.1186/s12957-020-1783-5

**Published:** 2020-01-21

**Authors:** Sebastian Hempel, Florian Oehme, Benjamin Müssle, Daniela E. Aust, Marius Distler, Hans-Detlev Saeger, Jürgen Weitz, Thilo Welsch

**Affiliations:** 1 Department of Visceral, Thoracic and Vascular Surgery, University Hospital Carl Gustav Carus, Technische Universität Dresden, Dresden, Germany; 2 Institute of Pathology, University Hospital Carl Gustav Carus, TU Dresden, Dresden, Germany

**Keywords:** Non-pancreatic periampullary cancer, Para-aortic lymph nodes, Pancreatoduodenectomy, Survival

## Abstract

**Background:**

Resection of the para-aortic lymph node (PALN) group Ln16b1 during pancreatoduodenectomy remains controversial because PALN metastases are associated with a worse prognosis in pancreatic cancer patients. The present study aimed to analyze the impact of PALN metastases on outcome after non-pancreatic periampullary cancer resection.

**Methods:**

One hundred sixty-four patients with non-pancreatic periampullary cancer who underwent curative pancreatoduodenectomy or total pancreatectomy between 2005 and 2016 were retrospectively investigated. The data were supplemented with a systematic literature review on this topic.

**Results:**

In 67 cases, the PALNs were clearly assigned and could be histopathologically analyzed. In 10.4% of cases (7/67), tumor-infiltrated PALNs (PALN+) were found. Metastatic PALN+ stage was associated with increased tumor size (*P* = 0.03) and a positive nodal stage (*P* < 0.001). The median overall survival (OS) of patients with metastatic PALN and non-metastatic PALN (PALN–) was 24.8 and 29.5 months, respectively. There was no significant difference in the OS of PALN+ and pN1 PALN patients (*P* = 0.834). Patients who underwent palliative surgical treatment (*n* = 20) had a lower median OS of 13.6 (95% confidence interval 2.7–24.5) months. Including the systematic literature review, only 23 cases with PALN+ status and associated OS could be identified; the average survival was 19.8 months.

**Conclusion:**

PALN metastasis reflects advanced tumor growth and lymph node spread; however, it did not limit overall survival in single-center series. The available evidence of the prognostic impact of PALN metastasis is scarce and a recommendation against resection in these cases cannot be given.

## Introduction

The treatment of choice for patients affected by periampullary cancer is a pancreaticoduodenectomy (PD) [1]. The spread of cancer to regional lymph nodes is an important prognostic factor after resection, independent of cancer histology [2]. Therefore, lymphadenectomy is considered a critical step of PD for cancer. While there is consensus about the major steps and extent of lymphadenectomy during PD, no strong recommendation is given for routine resection of para-aortic lymph node (PALN) station Ln16b1 [3]. These lymph nodes are located dorsal to the pancreas and do not belong to the regional lymph node stations. However, the Ln16b1 station is an important node in major lymphatic drainage [3]. Metastatic spread to this lymph node station is therefore classified as pM1 stage. Several studies—including our own recent work—concluded that tumor-bearing PALNs of pancreatic ductal adenocarcinoma (PDAC) were a predictor for poor overall survival [4–7]. The majority of available data investigates the impact of PALNs in patients with PDAC, but without non-pancreatic periampullary cancer. However, histopathologic phenotype seems to be an important and relevant prognostic factor affecting survival in patients with periampullary tumors [8]. The biological behavior of PALN metastasis of intestinal-type non-pancreatic periampullary cancer probably differs from that of the pancreaticobiliary types, such as PDAC, and has a better prognosis. There is little data on the outcome of PALN metastasis in non-pancreatic periampullary cancer. Thus, the primary aim of the present study was to investigate the subgroup of non-pancreatic periampullary cancer resections in our center with respect to PALN metastases and provide a systematic literature review of this topic.

## Methods

### Study design and patients

All patients who underwent partial pylorus-preserving PD (PPPD), classic PD (cPD), or total pancreatectomy (TP) for non-pancreatic periampullary cancer between January 2005 and December 2016 at the Department of Visceral, Thoracic and Vascular Surgery, University Hospital Carl Gustav Carus, Technische Universität Dresden, Germany were identified from a pancreatic database and retrospectively analyzed. Resection of lymph node Ln16b1 (limited PALN resection) was performed routinely. Nevertheless, the resected lymph node specimens could not be clearly assigned to PALN station Ln16b1 in all cases during the pathological work-up. This was because PALNs were not unequivocally labeled during every operation, thereby making it impossible to clearly differentiate them from peripancreatic lymph nodes in some cases. For the present study, only cases with clearly labeled PALNs were considered. These cases were divided into two groups: patients with a PALN-positive (PALN+) status (histopathological tumor invasion of at least one PALN) and patients without PALN metastasis (PALN–). The latter group was further stratified based on nodal status (pN0 or pN+). An additional comparative survival analysis was computed by taking into account all patients who underwent palliative surgery for metastatic or unresectable stage within the same observation time.

To evaluate tumor recurrence, follow-up data were collected during regular examinations in our outpatient clinic, as well as through phone calls or interviews with primary physicians. The clinical examination, an elevated carbohydrate antigen 19-9 (CA 19-9) level or imaging modalities (e.g., sonography, computed tomography (CT) or magnetic resonance imaging (MRI) scans) were used to detect tumor progression. The experimental protocol of the study was approved by the local ethics committee of the TU Dresden (decision number EK70022017).

### Operative technique

The principal operative techniques of PPPD and cPD have recently been described [9]. With respect to the lymphadenectomy, the PALNs (Ln16b1) were dissected dorsal to the pancreas during an extended Kocher maneuver. The PALNs were resected between the vena cava and aorta, including the ventral aspect of these vessels, beginning from the left renal vein to the superior margin of the inferior mesenteric artery. This PALN subgroup was routinely resected because it was included within the primary resection plane and the dissection was helpful for clear visualization of the superior mesenteric artery origin; other PALN stations such as Ln16b2 were not resected.

### Pathological assessment

After macroscopic examination, the resected para-aortic lymph node (PALN) specimens were histologically analyzed using hematoxylin and eosin slides without serial sectioning or immunohistochemical analyses for single tumor cells. Once tumor infiltration with desmoplastic reaction was detected, PALN metastasis was diagnosed.

### Systematic review

The systematic review was performed according to the Preferred Reporting Items for Systematic Reviews and Meta-Analysis (PRISMA) guidelines [10]. All published articles available in the MEDLINE, Embase, Web of Science, and Cochrane Library databases up to August 18, 2019 were screened. A search algorithm consisting of the corresponding MeSH terms was developed and adapted for the respective database. The search algorithm for the MEDLINE database (PubMed) is given in Table 5. The reference lists of all included articles were manually cross-checked in order to identify additional studies.

Two independent investigators (Sebastian Hempel and Benjamin Müssle) evaluated each article for inclusion. After removing the duplicates, all case reports and studies investigating inappropriate tumor entity were excluded. Only studies providing data on the results of overall survival after resection of non-pancreatic periampullary cancer with and without PALN metastasis were considered.

### Statistical analysis

The IBM SPSS 25 (SPSS Statistics v25, IBM Corporation, Armonk, New York) software package was used for statistical analysis and data plots. A value of *P* < 0.05 was considered statistically significant. Categorical and quantitative variables were analyzed using Fisher’s exact test and the unpaired *t*-test, respectively. All quantitative variables were expressed as median with interquartile range (IQR). Cox proportional hazards models were used to compute uni- and multivariate survival analyses. Patient age > 70 years, T and N stage, tumor grade, resection stage, neo- and adjuvant treatment, and PALN status were considered for univariate analysis. The quotient between tumor-infiltrated lymph nodes and resected lymph nodes was defined as the lymph node ratio (LNR) and stratified in LNR < 0.2 and ≥ 0.2. Variables found to be significant in univariate analysis were further used for multivariate testing. The number of positive and resected lymph nodes, tumor size, preoperative CA 19-9, and progression-free survival (PFS) time were considered for a multiple linear regression model. The overall survival and progression-free survival curves were determined using the Kaplan-Meier method. Differences between the survival curves were identified using the log-rank test. Overall survival (OS) was calculated as the period between the index operation until the date of death or the time of last contact (censored). Similarly, PFS was defined as the time to last follow-up contact without the progression of the tumor. The period from surgery to last patient contact or death of the patient was defined as the follow-up time.

## Results

### Patient cohort and histopathological tumor characteristics

One hundred sixty-four patients underwent pancreatoduodenectomy (PD) or total pancreatectomy (TP) for non-pancreatic periampullary cancer within the study period. In 67 cases (41%), the resected PALNs were separately labeled and histopathologically analyzed, whereas the resected lymph nodes could not be retrospectively assigned to the PALN compartment in the remaining cases. Tumor-infiltrated PALNs (PALN+) were proved in 10.4% of cases (7/67). In most cases, a pylorus-preserving pancreatoduodenectomy (PPPD) was performed (84%). No significant differences in the majority of standard patient and surgical characteristics were found between PALN+ and PALN− patients (Table 1). However, the PALN+ patients tended to have higher preoperative serum CA 19-9 levels. Furthermore, metastatic PALNs were always associated with regional lymph node infiltration. The significantly higher rate of nodal-positive status (pN1) and higher lymph node ratio reflect advanced lymphovascular spreading (Table 2). In addition, PALN metastases were significantly correlated with the number of metastatic regional lymph nodes (*P* < 0.001) and tumor size (*P* = 0.03) in a multiple linear regression model. In the most of PALN+ cases, only one PALN was tumor infiltrated (4 of 7 cases; range of metastatic PALN: 1–3). Histopathological reporting of the tumor subtype was available in 6 of the 7 PALN cases: 3 were intestinal, 2 were pancreaticobiliary, and 1 was of a mixed subtype.

**Table 1:** Patient and operative characteristics

Variable	PALN+	PALN–	*P*
Patients [*n*]	7	60	
Median age [years] (IQR)	67 (64–73)	71 (63–75)	0.798
Male sex [*n*/(%)]	2 (29)	35 (58)	0.228
Diabetes [*n*/(%)]	2 (29)	8 (13)	0.278
Weight loss [*n*/(%)]	5 (71)	27 (45)	0.245
Jaundice [*n*/(%)]	5 (71)	32 (53)	0.446
Median CA 19–9 [U/ml] (IQR)	166 (28–530)	46 (15–139)	0.583
Operation [*n*/(%)]			
PPPD	7 (100)	50 (83)	0.582
cPD	0	9 (15)	0.581
TP	0	1 (2)	1.000
Neoadj. therapy [*n*/(%)]	0	0	
Portal vein resection [*n*/(%)]	1 (14)	11 (18)	1.000
Median operative time [min] (IQR)	375 (313–473)	363 (298–439)	0.573
Adjuvant therapy [*n*/(%)]	4 (57)	17 (28)	0.193

Abbreviations: *PPPD*: Pylorus-preserving pancreaticoduodenectomy; *cPD*: Classic pancreatoduodenectomy; *TP*: Total pancreatectomy; *neoadj.*: Neoadjuvant; *IQR*: Interquartile range

**Table 2:** Histopathological tumor characteristics

Variable	PALN+	PALN–	*P*
Patients [*n*]	7	60	–
Tumor histology			
Distal bile duct carcinoma	5 (71)	30 (50)	0.430
Ampullary carcinoma	2 (29)	25 (40)	0.692
Carcinoma of duodenum	0	5 (8)	1.000
Tumor grade [*n*/(%)]			
G1/G2	2 (29)	24 (40)	0.697
G3/G4	5 (71)	35 (58)	0.692
Gx	0	1 (2)	1.000
pT			
T1/T2	2 (29)	23 (38)	0.704
T3/T4	5 (71)	37 (62)	
pN			
N0	0	29 (48)	**0.016**
N1	7 (100)	31 (52)	
pM			
M0	0	60 (100)	
M1	7 (100)		**0.001**
Resection status			
R0	7 (100)	55 (92)	1.000
R1	0	5 (8)	
Tumor size [mm] (IQR)	30 (22–35)	20 (15–30)	0.282
Median number of resected LN (IQR)	21 (18–25)	20 (13–25)	0.857
Median number of resected PALN (IQR)	5 (1–6)	4 (2–6)	0.556
LN ratio			
> 0.2	5 (71)	8 (13)	**0.002**
≤ 0.2	2 (29)	52 (87)	
PALN ratio			
> 0.2	6 (86)	0	**0.001**
≤ 0.2	1 (14)	60 (100)	

Abbreviations: *IQR*: Interquartile range; *LN*: Lymph node

*p* values < 0.05 are in boldface

No significant differences with regard to postoperative morbidity were detected in either subgroup (Table 3). However, the rate of postoperative pancreatic fistula (POPF) and delayed gastric emptying (DGE) was slightly higher in PALN– patients than in PALN+ patients (36% vs. 14%, 42% vs.14%). The overall 30-day mortality was 4.4% (3/67).

**Table 3:** Operative morbidity and mortality

Variable	PALN+	PALN–	*P*
Patients [*n*]	7	60	
Morbidity [*n*/(%)]	6 (86)	39 (60)	0.411
Diarrhea [*n*/(%)]	0	0	
Lymph fistula [*n*/(%)]	0	3 (5)	1.000
POPF [*n*/(%)]	1 (14)	21 (35)	0.411
PPH [*n*/(%)]	0	9 (15)	0.581
DGE [*n*/(%)]	1 (14)	25 (42)	0.233
SSI [*n*/(%)]	2 (29)	14 (23)	1.000
Abscess [*n*/(%)]	2 (29)	16 (26)	1.000
Thromboembolism [*n*/(%)]	1 (14)	4 (7)	0.434
Mortality			
30d [*n*/(%)]	0	3 (5)	1.000
60d [*n*/(%)]	0	3 (5)	1.000

Abbreviations: *POPF*: Postoperative pancreatic fistula B/C (classified according to Bassi); *PPH*: Postpancreatectomy hemorrhage; *DGE*: Delayed gastric emptying; *SSI*: Surgical site infection

### Survival analysis

**Fig. 1:**
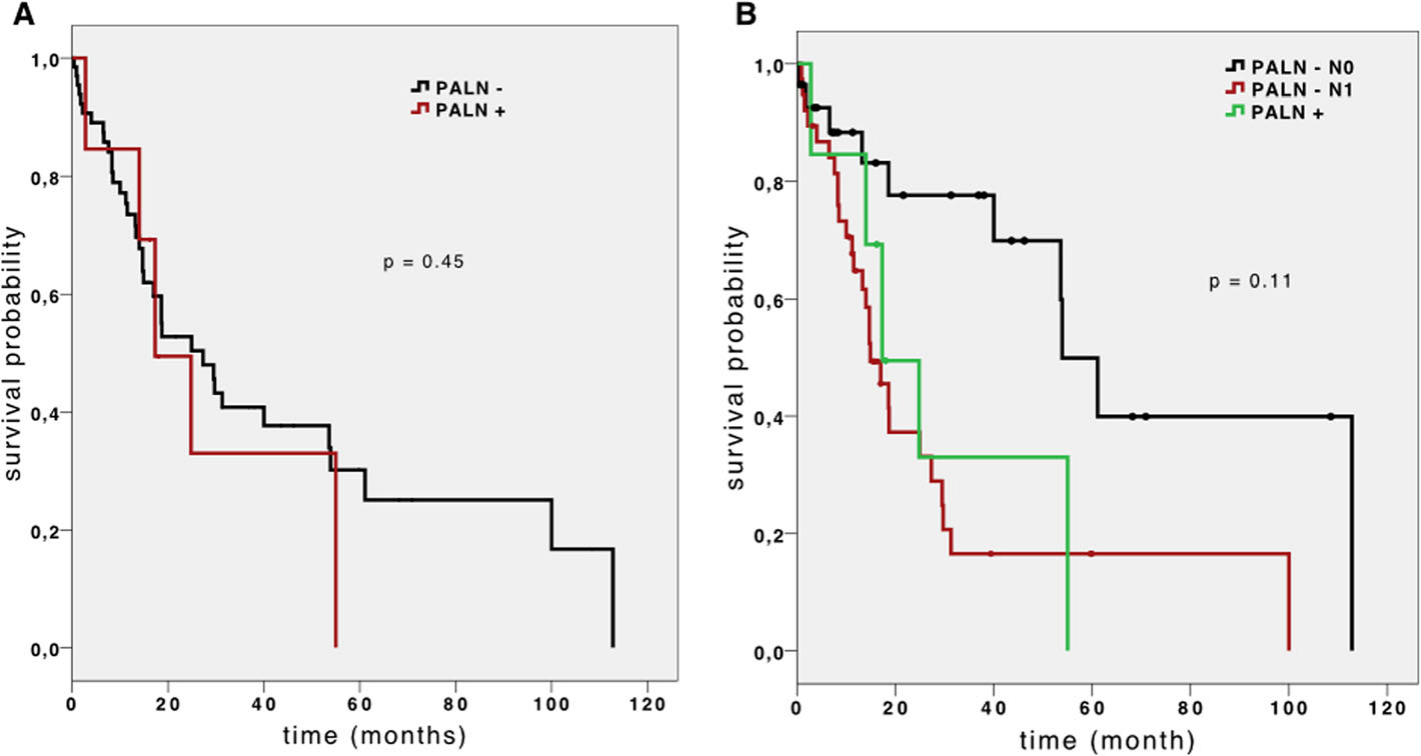
Overall survival of patients with para-aortic lymph node resection. **a** Overall survival of patients with para-aortic lymph node (PALN) resection (*n* = 67). Patient subgroups with PALN metastasis (PALN+, *n* = 7) and without PALN metastasis (PALN−, *n* = 60) were plotted. **b** Overall survival of patients with PALN resection separated into PALN+ (*n* = 7), PALN− pN0 (*n* = 29), and PALN− pN1 (*n* = 31) subgroups according to the regional lymph node status

**Fig. 2:**
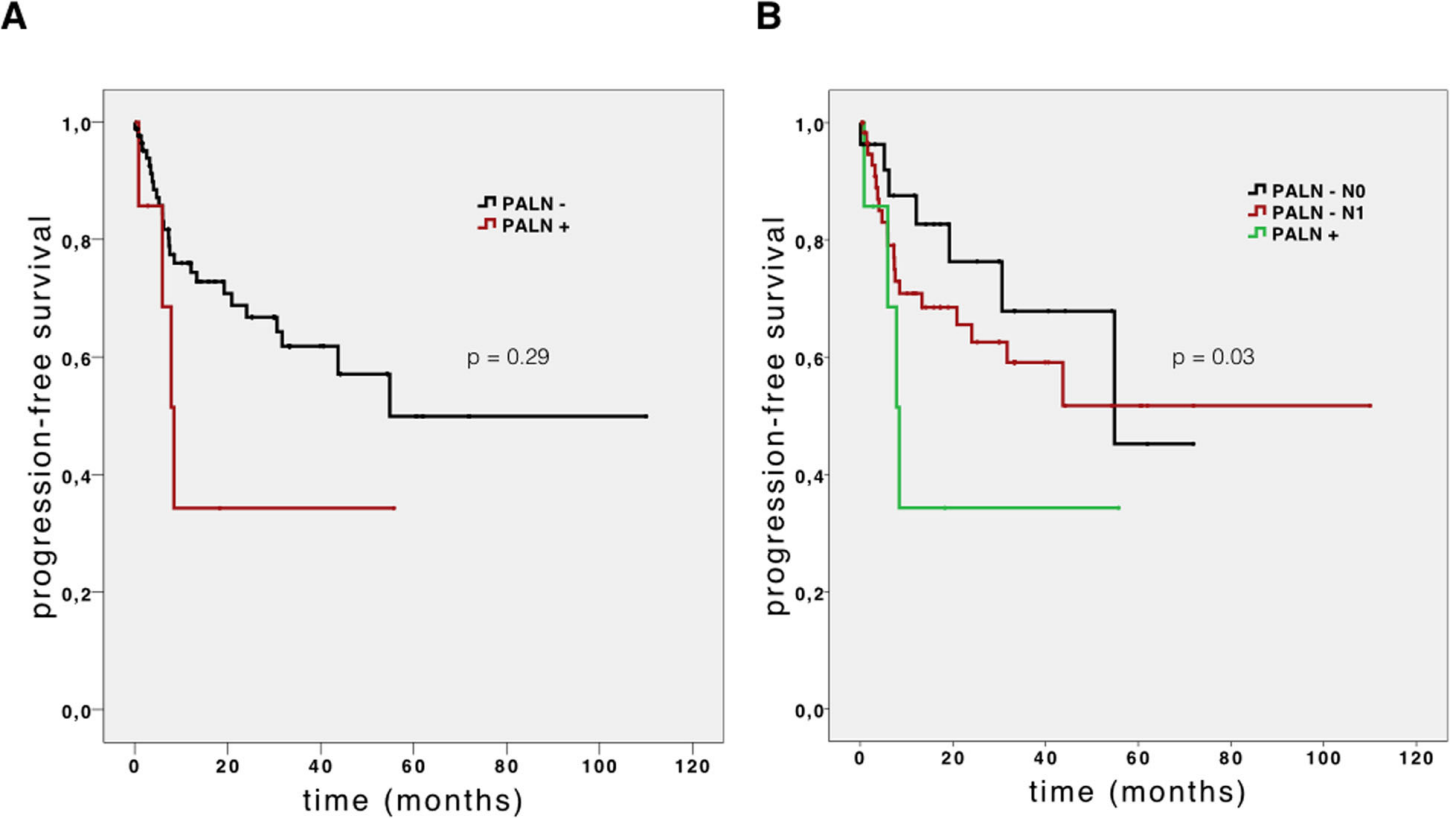
Progression-free survival of patients with para-aortic lymph node resection. **a** Progression-free survival of patients with para-aortic lymph node (PALN) resection (*n* = 67). Patient subgroups with PALN metastasis (PALN+, *n* = 7) and without PALN metastasis (PALN−, *n* = 60) were plotted. **b** Progression-free survival of patients with PALN resection separated into PALN+ (*n* = 7), PALN− pN0 (*n* = 29), and PALN− pN1 (*n* = 31) subgroups according to the regional lymph node status

The survival analysis was calculated using the Kaplan-Meier method with log-rank test. The median OS of PALN+ and PALN– patients was 24.8 (95% confidence interval [CI] 14.6–35.0) months and 29.5 (95% CI 8.2–50.8) months, respectively. Figure 1a shows the OS curves of the two subgroups. The median PFS in the PALN+ group was 8.3 (95% CI 5.5–11.1) months, whereas in the PALN– group it was 23.7 (95% CI 0–51.5). However, no statistical significance was calculated (*P* = 0.29). The curves of PFS of the two subgroups (PALN+ vs. PALN−) are shown in Fig. 2a. On univariate analysis, no significant correlation between PALN status and overall survival was found (*P* = 0.163). Only two significant factors affecting overall survival rates were identified on multivariate analysis (Table 4): nodal status (*P* < 0.01) and failure to receive adjuvant treatment (*P* < 0.01).

**Table 4:** Univariate and multivariate analysis

Variable	Univariate analysis	Multivariate analysis		
	*P*	OR	95% CI	*P*
Age > 70 years	0.07	1.138	0.350–3.701	0.83
pT-stage	0.08	1.804	0.707–4.606	0.217
pN-stage	**0.02**	6.124	1.377–27.239	**< 0.01**
Grading	1	1.255	0.414–3.806	0.69
Resection status	0.64	0.459	0.034–6.177	0.56
Adjuvant therapy	0.13	0.184	0.04–0.860	**< 0.01**
PALN status	0.163	0.756	0.099–5.804	0.79

Abbreviations: *CI*: Confidence interval; *IQR*: Interquartile range; *OR*: Odds ratio*LN*: Lymph node

*p* values < 0.05 are in boldface

In order to compare PALN with regional lymph node metastases, we generated two subgroups within the non-metastatic PALN cohort according to their nodal stage into 29 cases without regional lymph node metastasis and 31 nodal-positive (pN1) cases. In the pN0 subgroup, we noticed a significantly longer (*P* = 0.011) median overall survival (53.9 months, 95% CI 43.3–64.4) than that of the pN1 subgroup (OS 17.0 months, 95% CI 11.7–22.2) or PALN+ cohort. Figure 1b shows the OS curves of these three subgroups. On the other hand, there was no significant difference in median OS of the PALN– pN1 and PALN+ (*P* = 0.834) subgroups. Furthermore, the PALN– pN0 subgroup also had a significantly longer PFS (*P* = 0.033). In Fig. 2b, the PFS curves of these three subgroups are shown.

We further analyzed the survival probability of PALN+ patients and all patients treated palliatively within the study period. Twenty patients who underwent surgical exploration or palliative bypass surgery for unresectable or metastatic, non-pancreatic periampullary cancer were identified. The median OS of these 20 patients was 13.6 (95% CI 2.7–24.5) months and significantly shorter than that of the PALN+ subgroup (*P* = 0.03).

### Systematic literature review

Due to the limited cohort size of patients with non-pancreatic periampullary cancers and PALN data, we conducted a systematic electronic literature search (Table 5).

**Table 5:** Search strategy and results for the systematic literature review as used for MEDLINE

Search	Query	Items found
Population		
#1	Distal AND (bile duct cancer OR cholangiocarcinoma) OR “Periampullary cancer” OR “peri-ampullary” OR adenocarcinoma AND (bile duct OR ampulla) OR “ampullary cancer”	14727
Intervention		
#2	Pancreatoduodenectomy OR pancreaticoduodenectomy OR duodenopancreatectomy OR pancreatectomy OR Whipple OR “Pancreatic head resection” OR “surgical resection” OR resection	291546
End points		
#3	(Interaortocaval OR para-aortal OR para-aortic) AND (“lymph nodes” OR metastas* OR lymphadenectomy) OR PALN	3528
Synthesis		
#4	#1 AND #2 AND #3	40

**Fig. 3:**
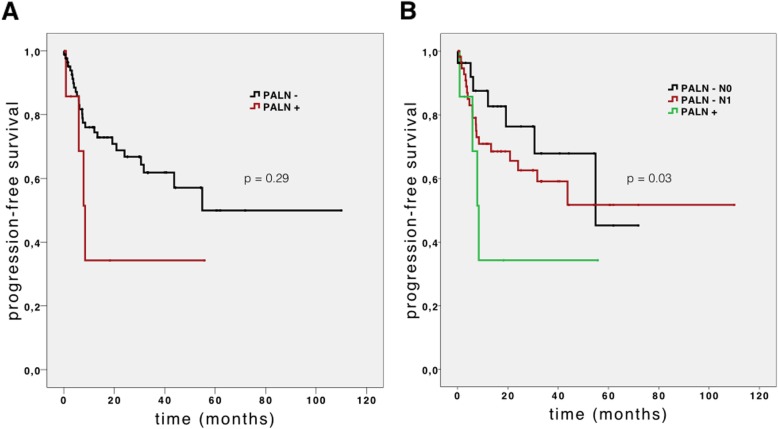
PRISMA flow diagram of the systematic literature search

A total of 87 abstracts were identified. After a detailed assessment according to the inclusion criteria, only 2 studies could be considered for further data extraction (Fig. 3). These 2 studies combined with our present data provided data on 23 patients with PALN metastases after resection of non-periampullary cancers. In 2004, Yoshida et al. reported on 6 of 36 PALN-positive patients, with a median overall survival of 19 months [11]. In 2016, Hafeez Bhatti et al. reported on 10 of 40 patients with involved PALN and a median overall survival of 17.5 months [12] (Table 6). The mean survival of the 23 patients with PALN metastases was 19.8 months.

**Table 6:** Results of the systematic literature review

Reference	Year	Study design	Analyzed cases (*n*)	PALN+ cases (*n*)	Median OS (months) PALN+ PALN−	
Yoshida et al .[11]	2004	Retrospective	36	6	19.0	n/a
Hafeez Bhatti et al .[12]	2016	Retrospective	40	10	17.5	17.0
Present study	2019	Retrospective	67	7	24.8	29.5

Abbreviations: *PALN*: Para-aortic lymph node; *OS*: Overall survival

## Discussion

Regarding on the management of PALN, no consensus has been reached so far [3]. Extended lymphadenectomy or PALN resection during oncological PD is still an ongoing discussion and area of controversy. The present study demonstrates that approximately 10% of non-pancreatic periampullary cancer cases harbor PALN metastases at the time of resection. Previous studies have reported comparable sample sizes (Table 7) with a higher incidence of PALN metastasis (17–25%) [11, 12]. In the study by Connor et al., the incidence of PALN metastases in non-pancreatic periampullary cancer (only distal bile duct carcinoma considered) was significantly higher at 55% [13].

**Table 7:** Incidence of PALN metastases in non-pancreatic periampullary cancer

Reference	Year	Study design	Analyzed cases (*n*)	PALN+ cases [*n*/(%)]	Population	Histology
Yoshida et al .[11]	2004	Retrospective	36	6 (17)	A	DBC
Hafeez Bhatti et al .[12]	2016	Retrospective	40	10 (25)	A	Non-pancreatic
Connor et al .[13]	2004	Retrospective	11	6 (55)	E	DBC
Nappo et al .[6]	2015	Retrospective	49	3 (6)	E	AC, DBC
Murakami et al .[14]	2011	Retrospective	63	9 (14)	A	AC, DBC
Present study	2019	Retrospective	67	7 (10)	E	Non-pancreatic

Abbreviations: *A*: Asian; *AC*: Ampullary cancer; *DBC*: Distal bile duct cancer; *E*: European

Yoshida et al., reported a median overall survival of 19 months in cases of PALN metastasis; however, only cases of distal bile duct involvement were investigated [11]. In 2016, Hafeez Bhatti et al. demonstrated comparable survival data between PALN+ (17.5 months) and PALN− subgroups (17 months). In our analysis, the patients with and without PALN metastases survived 24.8 and 29.5 months, respectively. These findings indicate that PALN+ and PALN− status may lead to similar survival after resection. However, there seems to be a difference in median overall survival and the frequency of PALN metastasis in non-pancreatic periampullary cancer between Asian and European populations [11, 12].

The relevance of the extent of lymphadenectomy in resected periampullary cancer has been described by some previous studies [15–17]. In general, the lymph node ratio rather than lymph node status was identified as a prognostic factor. Recently, Liu et al. published a definition of an extended minimum level of at least 16 lymph nodes in non-pancreatic periampullary cancer resections [18].

A comparison of the median survival duration of patients with palliative surgery (13.6 months) or PALN+ resection (24.8 months) showed that patients in the latter subgroup demonstrated a longer survival of approximately 10 months. Thus, PALN resection (Ln16b1) in cases of non-pancreatic periampullary cancer may provide an advantage for patients.

A recent study reported the impact of the histopathologic phenotype of periampullary cancer on survival and response to therapy [8]. The findings highlight the clear need to assess tumor biology and growth, treatment regimen and the prognosis of peripancreatic tumors with respect to genetic, molecular or histologic subtypes in the future. Based on the findings of Williams et al., the intestinal phenotype is associated with a better outcome than the pancreatobiliary phenotype. In the present study, the following subtypes were found in 6 of the 7 PALN cases: 3 intestinal, 2 pancreaticobiliary, and 1 mixed type. Therefore, we could not prove the influence of histopathologic phenotype on PALN metastasis.

A recent meta-analysis explored the prognostic value of lymph node metastases in pancreatic and periampullary cancer [19]. Most of the included studies focused on the impact of PALN metastasis; however, isolated survival data in cases of PALN metastases detected during surgical resection for non-pancreatic periampullary cancer were not provided.

The results and conclusions of the present analysis are significantly limited by the small number of available data sets (7 patients with PALN metastases). PALN resection was routinely performed at our institution; however, a definite allocation of the resected lymph nodes to the Ln16b1 station (PALN) was only possible in 67 of the 164 cases. This was due to the fact that PALNs were not routinely labeled at the time of resection. Nevertheless, the tumor biology of intestinal-type tumors in the periampullary region is less aggressive than pancreatic cancer. In addition, the little available data must be analyzed to determine the optimal operative treatment of this cancer subtype. All previous studies reporting on periampullary cancers and PALN metastases investigated a similar number of cases with comparable survival data [11, 12]. Contrary to other studies on PALN resection, the present study explicitly analyzed cases of non-pancreatic periampullary cancer.

## Conclusion

The present study indicates that patients with non-pancreatic periampullary cancers and PALN metastases (pM1-LYM) may have a prognosis similar to that of patients without PALN metastases, however, this observation is based on very few cases. This is in contrast to pancreatic cancer patients, who have a worse survival in cases of PALN metastases [4–7]. Although tumor infiltration of PALNs is an indicator of advanced tumor growth and regional lymph node metastasis, patients with non-pancreatic periampullary cancer and suspected or proven PALN metastases may benefit from aggressive tumor resection. This management approach could be further supported by an improved current neo- or adjuvant chemotherapeutic regimen, e.g., gemcitabine/capecitabine or Folfirinox. In the future, subtype characterization of the tumor entities in the periampullary region (e.g., intestinal and pancreaticobiliary subtypes) will probably serve as a guide for resection strategies. This study demonstrates the need for differential analysis of peripancreatic tumor entities in the future.

## Data Availability

The datasets that were analyzed during the current study are available from the corresponding author upon reasonable request.

## Abbreviations

BMI: Body mass index
cPD: Classic pancreaticoduodenectomy
CT: Computed tomography
DGE: Delayed gastric emptying
ISGPF: International Study Group for Pancreatic Fistula
LNR: Lymph node ratio
MRI: Magnetic resonance imaging
OS: Overall survival
PALNs: Para-aortic lymph nodes
PD: Pancreaticoduodenectomy
PDAC: Pancreatic ductal adenocarcinoma
PFS: Progression-free survival
POPF: Postoperative pancreatic fistula
PPH: Postpancreatectomy hemorrhage
PPPD: Pylorus-preserving pancreatoduodenectomy
SSI: Surgical site infection
TP: total pancreatectomy
